# Using Imiquimod-Induced Psoriasis-Like Skin as a Model to Measure the Skin Penetration of Anti-Psoriatic Drugs

**DOI:** 10.1371/journal.pone.0137890

**Published:** 2015-09-10

**Authors:** Yin-Ku Lin, Sien-Hung Yang, Chin-Chuan Chen, Hsiao-Ching Kao, Jia-You Fang

**Affiliations:** 1 School of Traditional Chinese Medicine, Chang Gung University, Kweishan, Taoyuan, Taiwan; 2 Department of Traditional Chinese Medicine, Chang Gung Memorial Hospital at Keelung, Keelung, Taiwan; 3 Department of Traditional Chinese Medicine, Chang Gung Memorial Hospital at Linkou, Taoyuan, Taiwan; 4 Molecular Genetics Laboratory, Graduate Institute of Natural Products, Chang Gung University, Kweishan, Taoyuan, Taiwan; 5 Pharmaceutics Laboratory, Graduate Institute of Natural Products, Chang Gung University, Kweishan, Taoyuan, Taiwan; 6 Chinese Herbal Medicine Research Team, Healthy Aging Research Center, Chang Gung University, Kweishan, Taoyuan, Taiwan; 7 Research Center for Industry of Human Ecology, Chang Gung University of Science and Technology, Kweishan, Taoyuan, Taiwan; CNRS-University of Toulouse, FRANCE

## Abstract

**Objective:**

Psoriasis is a chronic inflammatory skin disease and topical therapy remains a key role for treatment. The aim of this study is to evaluate the influence of psoriasis-like lesions on the cutaneous permeation of anti-psoriatic drugs.

**Methods:**

We first set up imiquimod-induced dermatitis in mice that closely resembles human psoriasis lesions. The development of the lesions is based on the IL-23/IL17A axis for phenotypical and histological characteristics. Four drugs, 5-aminolevulinic acid (ALA), tacrolimus, calcipotriol, and retinoic acid, were used to evaluate percutaneous absorption.

**Results:**

The most hydrophilic molecule, ALA, revealed the greatest enhancement on skin absorption after imiquimod treatment. Imiquimod increased the skin deposition and flux of ALA by 5.6 to 14.4-fold, respectively, compared to normal skin. The follicular accumulation of ALA was also increased 3.8-fold. The extremely lipophilic drug retinoic acid showed a 1.7- and 3.8-fold increase in skin deposition and flux, respectively. Tacrolimus flux was enhanced from 2 to 21 μg/cm^2^/h by imiquimod intervention. However, imiquimod did not promote skin deposition of this macrolide. The lipophilicity, but not the molecular size, dominated drug permeation enhancement by psoriatic lesions. The in vivo percutaneous absorption of ALA and rhodamine B examined by confocal microscopy confirmed the deficient resistance of epidermal barrier for facilitating cutaneous delivery of drugs via psoriasis-like skin.

**Conclusion:**

We established the topical delivery profiles of anti-psoriatic drugs via imiquimod-treated psoriasis-like skin.

## Introduction

Psoriasis is a chronic inflammatory skin disease characterized with epidermal hyperplasis, leukocyte infiltration, inflammation, and increased vascularity in dermis [1]. It is well-known that skin can be functionally and structurally altered by psoriasis. It is critical to document the absorption level from drugs penetrating into/through such diseased skin to assess the risk of over-absorption. In addition to the stratum corneum (SC), the role of the epidermal junctions in viable epidermis as a significant barrier to drug permeation cannot be ignored [2,3]. Despite many reports linking the capacity of the SC in drug diffusion, few studies have been conducted exploring the role of the epidermis in relation to drug absorption.

Drug permeation by topical products is generally examined via normal or intact skin; however, topical products are usually applied to diseased skin. Some efforts have been done previously for comparing the drug penetration between intact and damaged skins. The approaches for producing damaged skin for permeation evaluation include heating, delipidization, tape stripping, abrasion, and photoaging [4–6]. The impairment of skin barrier function is described for psoriasis [7]. The penetration barrier deterioration may be expected to accelerate drug delivery into the skin, augmenting the risk of over-absorption. On the other hand, the increased epidermal thickness by abnormal keratinization in psoriasis creates a longer pathway through which the drugs have to diffuse [8]. There is little literature that discusses the extent to which psoriasis actually modulates drug absorption; therefore, it is our aim to compare cutaneous delivery profiles of anti-psoriatic drugs between normal and psoriasis-like skin.

We employed a mouse model to mimic the aspects of psoriasis. Topical application of imiquimod, a ligand of toll-like receptor (TLR) 7 and 8, has been recently reported to induce psoriasis-like dermatitis [9]. Four drugs, 5-aminolevulinic acid (ALA), tacrolimus, calcipotriol, and retinoic acid, frequently used to treat psoriasis vulgaris were selected as the model permeants in this study.

## Materials and Methods

### Materials

ALA, retinoic acid, and rhodamine B were purchased from Sigma-Aldrich (St. Louis, MO, USA). Tacrolimus was a standard reference from U.S. Pharmacopeia (Rockville, MD, USA). Calcipotriol was supplied by Cayman (Ann Arbor, MI, USA). The 5% imiquimod cream (Aldara) was a gift from 3M Pharmaceuticals (Leicestershire, UK).

### Animals

Female BALB/cByJNarl mice at 7 weeks old were supplied by National Laboratory Animal Center (Taipei, Taiwan). This study was carried out in strict accordance with the recommendations in the Guidelines for the Care and Use of Laboratory Animals of Chang Gung University of Science and Technology. Ethical issues with animal experiments complied with Directive 86/109/EEC from the European Commission. The protocol was approved by the Institutional Animal Care and Use Committee of Chang Gung University of Science and Technology (Permit Number: 2013–005). The alfalfa-free food (#5058, Labdiet, Framingham, MA, USA) and water were given ad libitum. All efforts were made to minimize suffering.

### Induction of psoriasis-like skin

This method was modified from the protocol by van der Fits [10]. The mouse was shaved on the dorsal skin. The imiquimod cream with a dose of 62.5 mg was topically administered on the mouse back every day for 8 consecutive days. The sham control mouse was treated similarly with the vanishing cream base (United States Pharmacopeia). The ingredients of vanishing cream vehicle were stearic acid (25%, w/w), triethanolamine (1.35%), lanolin (4%), propylene glycol (5%), methyl paraben (0.18%), propyl paraben (0.02%), and water (64.45%).

### Gross observation

The gross imaging of mouse skin treated with or without imiquimod cream was observed using a handheld digital microscope (Mini Scope-V, M&T Optics, Taipei, Taiwan). A magnification of 200x was used for imaging capture.

### Physiological parameters

Immediately after an 8-day application of imiquimod, the mouse skin was examined for physiological parameters, including transepidermal water loss (TEWL), erythema, and skin surface pH. A Tewameter (TM300, Courage and Khazaka, Köln, Germany) was employed for measuring TEWL (g/m^2^/h). A spectrocolorimeter (CD100, Yokogawa, Tokyo, Japan) was used to quantify cutaneous erythema. The pH was determined by Skin-pH-Meter PH905 (Courage and Khazaka).

### Histological analysis

The dorsal skin was excised from the mouse after sacrifice. The skin species were immersed in a 10% buffered formaldehyde using ethanol, embedded in paraffin wax, and sliced at a thickness of 3 μm. The samples were stained by hemoxylin and eosin (H&E) and imaged under light microscopy (IX81, Olympus, Tokyo, Japan). For immunohistochemistry (IHC) observation, the biopsies were incubated for 1 h at room temperature with primary antibodies against the following cells or antigens: CD3^+^ and β-catenin. The skin sections stained with CD3^+^ and β-catenin were visualized by light microscopy. Immunoreactivity was detected with an Alexa Fluor 594 goat anti-rabbit immunoglobulin antibody (Invitrogen, Carlsbad, CA, USA).

### Bio-Plex cytokine measurement

The skin samples were rinsed by cell wash buffer. A 500 mM phenylmethylsulfonyl fluoride in cell lysis buffer (0.5 ml) was prepared and added into test tubes with skin samples. The probe-type sonicator (GM70, Bandelin Electronic, Berlin, Germany) was used to homogenize the mixture for 10 s. Subsequently the mixture was freezed by liquid nitrogen. After thawing at room temperature, the mixture was homogenized by the sonicator for 10 s again. The sample was then centrifuged at 4500 x*g* for 4 min. The supernatant was collected and the cytokine concentration was quantified by using Bio-Plex Cytokine Assay Kit (Bio-Rad, Hercules, CA, USA) according to the method described previously [11]. The prepared sample was analyzed by Bio-Plex suspension array system (Luminex 100, Bio-Rad). The cytokines analyzed by Bio-Plex were interleukin (IL)-1β, IL-12, granulocyte macrophage colony-stimulating factor (GM-CSF), interferon (IFN)-γ, and tumor necrosis factor (TNF)-α.

### Quantitative real-time PCR (qRT-PCR)

Total RNA was extracted with Trizol (Invitrogen) and cDNA was synthesized by reverse transcription using iScript cDNA Synthesis Kit (Bio-Red). The qRT-PCR was performed using iQ SYBR Green Supermix (Bio-Red) according to the manufacturer’s instructions. The housekeeping gene GAPDH was used as an internal control and gene expression was calculated using comparative the threshold cycle method. The following primers were used to analyze target mRNA expression: TNF-α: 5’-CCCTCACACTCAGATCATCTTCT-3’ (forward) and 5’-GCTACGACGTGGGCTACAG-3’ (reverse); IL-17A: 5’-GCTCCAGAAGGCCCTCAG-3’ (forward) and 5’-CTTTCCCTCCGCATTGAC-3’ (reverse); IL-23: 5’-CCCGTATCCAGTGTGAAGATG-3’ (forward) and 5’-CCCTTTGAAGATGTCAGAGTCA-3’ (reverse); filaggrin: 5’-GTTTCCAAACACATGGATCAAAT-3’ (forward) and 5’-TTTGAATCTTGTTGGTGTCTGTG-3’ (reverse); involucrin: 5’-AAACTTGGTGAGCCAGAATTACA-3’ (forward) and 5’-CCTTTCCAGTTGTTTACCCTTCT-3’ (reverse). GAPDH was an internal control: 5’-AGCTTGTCATCAACGGGAAG-3’ (forward) and 5’-TTTGATGTTAGTGGGGTCTCG-3’ (reverse).

### Skin penetration of anti-psoriatic drugs

The experiment was conducted with Franz diffusion cell (Chin-Fa Glass, Hsinchu, Taiwan). The excised skin with or without imiquimod treatment was mounted between the donor and receptor compartments with SC facing upward into the donor. The receptor medium was pH 7.4 buffer (5.5 ml) for ALA to maintain a sink condition. The receptor contained 30% ethanol in pH 7.4 buffer for the permeation of tacrolimus, calcipotriol, and retinoic acid. The donor (0.5 ml) was loaded with ALA (6.4%, w/v, 32 mg in donor), tacrolimus (0.3%, 1.5 mg in donor), calcipotriol (0.06%, 0.3 mg in donor), or retinoic acid (0.1%, 0.5 mg in donor). The pH 5 buffer, 30% propylene glycol (PG)/pH 7.4 buffer, 30% ethanol/water, and 30% PG/water was employed as donor vehicle for ALA, tacrolimus, calcipotriol, and retinoic acid, respectively. The effective diffusion region for permeants was 0.785 cm^2^. The receptor temperature and stirring rate of the stirrer in receptor were kept at 37°C and 600 rpm, respectively. At determined intervals, a 300-μl medium was withdrawn from the receptor. After a 24-h application, the skin was removed from the Franz cell. The permeant amount within the cutaneous reservoir was extracted by 0.1 N HCl for ALA and methanol for the other drugs.

All samples were analyzed by high-performance liquid chromatography (HPLC) for estimating drug concentration in skin and receptor. The HPLC setup (Hitachi, Tokyo, Japan) for ALA, calcipotriol, and retinoic acid was described in our previous studies [12–14]. For tacrolimus, a reverse-phase C18 column (LiChrospher, Merck, Darmstadt, Germany) was used as the stationary phase. A column oven set at 50°C was employed to heat the column. The mobile phase consisted of acetonitrile/double-distilled water (70: 30) for tacrolimus assay. The flow rate and detection wavelength was set at 1.1 ml/min and 210 nm, respectively.

### Hair follicle uptake

Differential stripping and cyanoacrylate skin surface casting were utilized to detect the content of drugs in hair follicles [15]. Subsequent to stripping the SC by the adhesive tape, a follicular cast was prepared. A drop of superglue was added on a glass slide, which was pressed onto the surface of SC-stripped skin. The cyanoacrylate polymerized, and the slide was expelled with one quick movement after 5 min. The superglue remaining on the slide was scraped off and positioned in a tube with 2 ml methanol. The tube was shaken for 3 h. The final product was vacuumed to evaporate methanol. Mobile phase or water was added to dissolve the residuals for HPLC assay.

### In vivo skin distribution of ALA and rhodamine B

A glass cylinder with a hollow area of 0.785 cm^2^ was attached to mouse back by superglue. An aliquot of 0.2 ml pH 5 buffer containing ALA (0.64%) or 30% PG/water containing rhodamine B (0.03%) as the permeant was pipette into the cylinder. The application period was 6 h. The animal was then sacrificed, and the treated skin area was excised. A confocal laser scanning microscopy (CLSM, TCS SP2, Leica, Wetzlar, Germany) was used to observe horizontal section of the skin. The thickness of skin was scanned at ~5 μm increments via *z*-axis. Images were taken by summing 15 fragments at different depths from skin surface.

### Statistical analysis

Statistical analysis of differences between the groups was performed using Kruskal-Wallis test. The post hoc test for checking individual differences was Dunn’s test. A 0.05 level of probability (*p* < 0.05) was taken as the level of significance.

## Results

### Gross observation and physiological parameters

**Fig 1:**
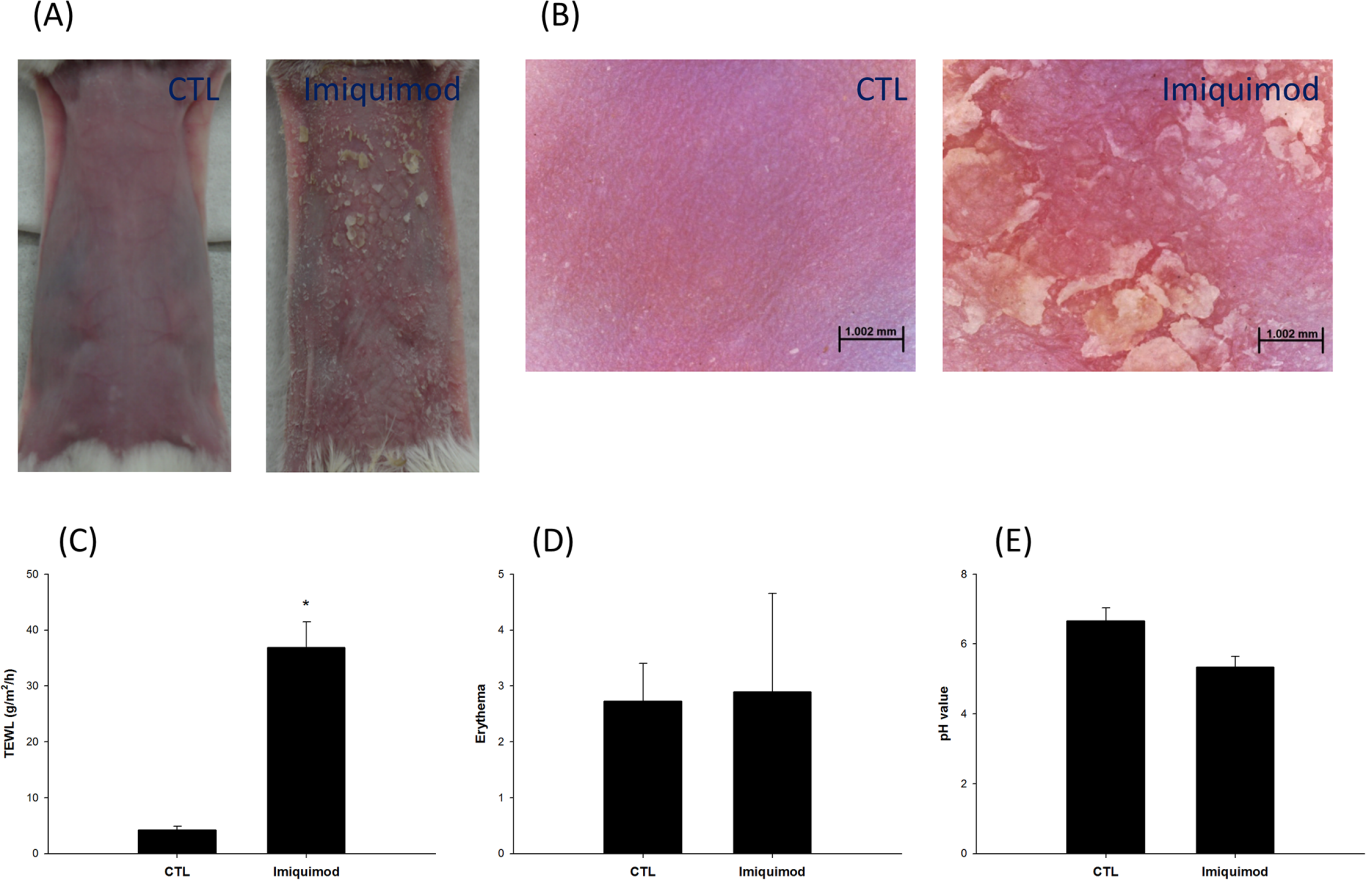
Gross observation and physiological parameters of mouse skin treated with and without imiquimod cream: (A) the gross imaging by digital camera; (B) the gross imaging by handheld digital microscope; (C) transepidermal water loss (TEWL); (D) erythema; and (E) skin surface pH value. The control group corresponds to the mice treated with the vehicle. The data of physiological assessment are presented as the mean of six experiments±S.D. *, *p* < 0.05 between control and imiquimod groups.

We first compared the macroscopic visualization and physiological condition between control and imiquimod-induced psoriasis-like skin. Fig 1A depicts the macroscopic images of the hairy mouse skin. At this level, imiquimod-induced psoriasis-like skin had scaly lesions and slight erythema. The close-up images of the control and imiquimod-induced psoriasis-like surfaces are illustrated in Fig 1B. The control skin was intact with furrows in the skin surface. The level of sebum on the skin surface for the imiquimod-treated group was reduced which caused the skin to appear darker with significant scaling and redness. The physiological changes caused by imiquimod were determined based on bioengineering approaches, including TEWL, erythema, and skin pH. As shown in Fig 1C, the TEWL for the control group was 4.24 g/m^2^/h. The imiquimod-treated group had a significantly higher (*p* < 0.05) level of TEWL (36.90 g/m^2^/h). TEWL is associated with the degree of barrier damage. There was a slight increase in erythema in the imiquimod-treated skin compared to control skin, however, this difference was not statistically significant (*p* > 0.05) (Fig 1D). In addition, the pH value of skin surface for the imiquimod-treated group showed a significant decrease (p < 0.05) (Fig 1E).

### Histological analysis

**Fig 2:**
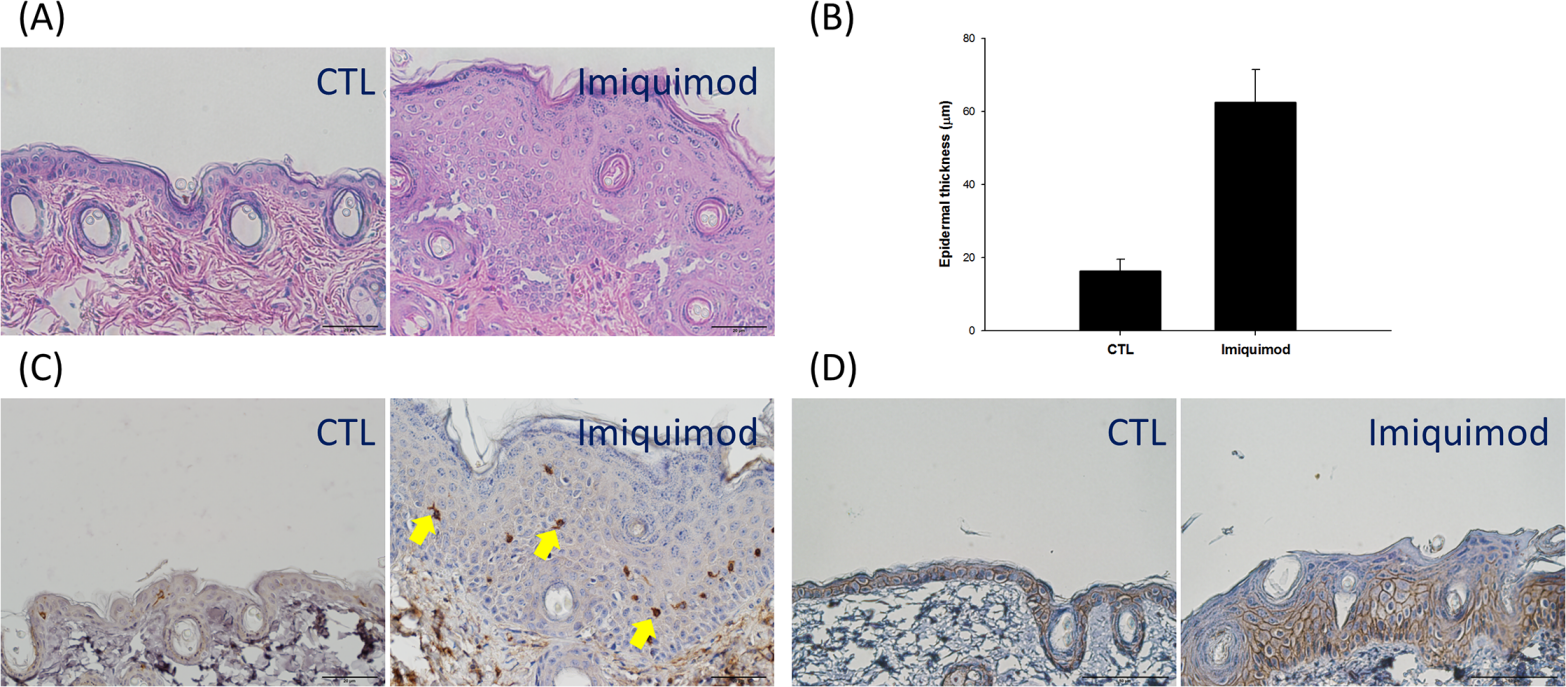
Histological and immunohistochemical examination of mouse skin treated with and without imiquimod cream: (A) hemoxylin and eosin (H&E) staining; (B) the epidermal thickness measured by H&E staining; (C) CD3^+^ T cell staining; and (D) β-catenin. The control group corresponds to the mice treated with the vehicle.

Fig 2 is the results from H&E and IHC staining of the mouse skin treated by imiquimod to investigate the changes between groups. As shown in the left panel of Fig 2A, H&E histology exhibits an intact structure on the control skin treated with vanishing cream. We had also determined TEWL, erythema, gross appearance, and H&E staining of the blank skin (the skin without any treatment). All profiles of blank skin approximated the results of control vehicle (*p* > 0.05) (see Table and Fig in S1 Text). At the microscopic level of imiquimod-treated skin, there was an increase in the epidermal thickness and desquamation. The increase in keratinocyte in the basal layer led to hyperproliferation and acanthosis. The epidermal thickness in the imiquimod-treated group was approximately four times thicker than the control group after eight days of treatment (Fig 2B). The lymphocyte infiltration for the imiquimod-treated group was greater than the control group based on the CD3^+^ T cells in the epidermis (Fig 2C). We evaluated the pericellular staining of β-catenin expression in the epidermal layers to determinate whether imiquimod affects epidermal junction since β-catenin is a biomarker of adherens junctions. This is detected by a distinct line in the intercellular regions of intact skin (the right panel of Fig 2D). A selective loss of β-catenin occurred in the focal regions of psoriasis-like skin, especially in the upper epidermis. Abundant amounts of β-catenin remained in the lower epidermis after being treated with imiquimod.

### Bio-Plex cytokine measurement

The mediators of inflammation such as cytokines are important to govern psoriatic pathophysiology. We used the Bio-Plex technique to determine the cytokine levels in the control and imiquimod-treated skin. As shown in Table 1, imiquimod significantly promotes (*p* < 0.05) the production of IL-1β, GM-CSF, and TNF-α by 2.5-, 2.3-, and 1.7-fold, respectively. Imiquimod intervention did not alter (*p* > 0.05) the levels of IL-12 and IFN-γ.

**Table 1:** Cytokine levels in normal and psoriatic skins.

Cytokine (pg/ml)	Control^a^	Psoriasis-like
IL-1β	122.19±16.96	307.02±12.56
IL-12	74.11±16.00	88.35±18.60
GM-CSF	30.56±6.20	69.48±17.93
IFN-γ	23.06±4.10	30.97±9.27
TNF-α	573.62±112.56	950.45±161.63

^a^ The control group corresponds to the mice treated with the vehicle.

Each value represents the mean and S.D. (*n* = 6).

### Quantitative real-time PCR (qRT-PCR)

**Fig 3:**
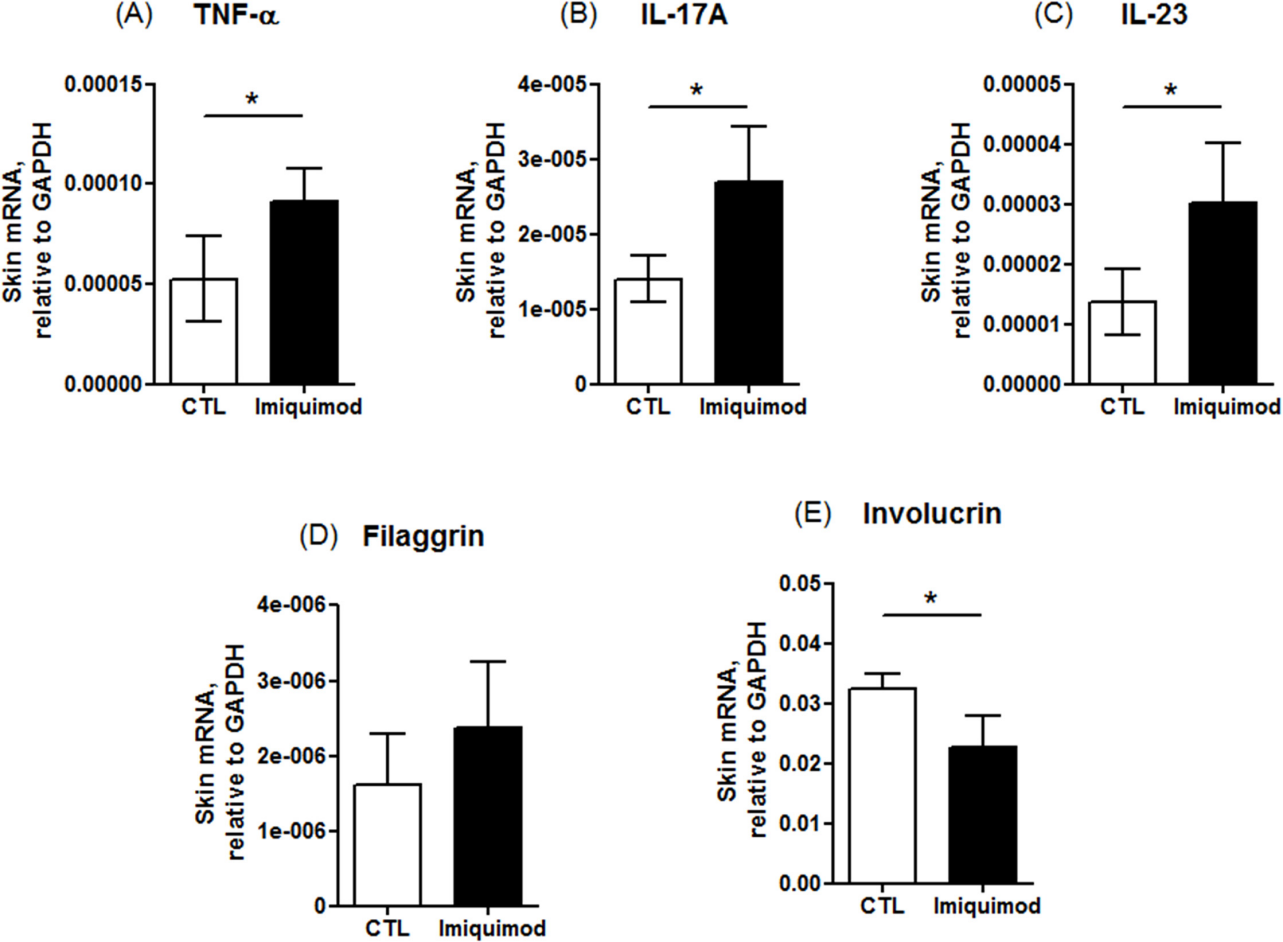
The mRNA levels of cytokines and differentiation markers in mouse skin treated with and without imiquimod cream: (A) TNF-α; (B) IL-17A; (C) IL-23; (D) filaggrin; and (E) involucrin. The control group corresponds to the mice treated with the vehicle. The data are presented as the mean of six experiments±S.D. *, *p* < 0.05 between control and imiquimod groups.

Some cytokines were also determined by the mRNA levels using qRT-PCR analysis as shown in Fig 3. Both Bio-Plex and qRT-PCR demonstrated the up-regulation of TNF-α by imiquimod (Fig 3A). Imiquimod-treated lesions increased the mRNA expression of either IL-17A or IL-23 (Fig 3B and 3C). The qRT-PCR was also utilized to check the effect of imiquimod-treated skin on gene expression involved in epidermal barriers. The qRT-PCR assay of mRNA from imiquimod-treated skin indicated a comparable filaggrin as compared to control skin (Fig 3D). In the psoriasis-like skin, involucrin mRNA expression was significantly reduced compared to the control skin (Fig 3E).

### Skin penetration of anti-psoriatic drugs

**Fig 4:**
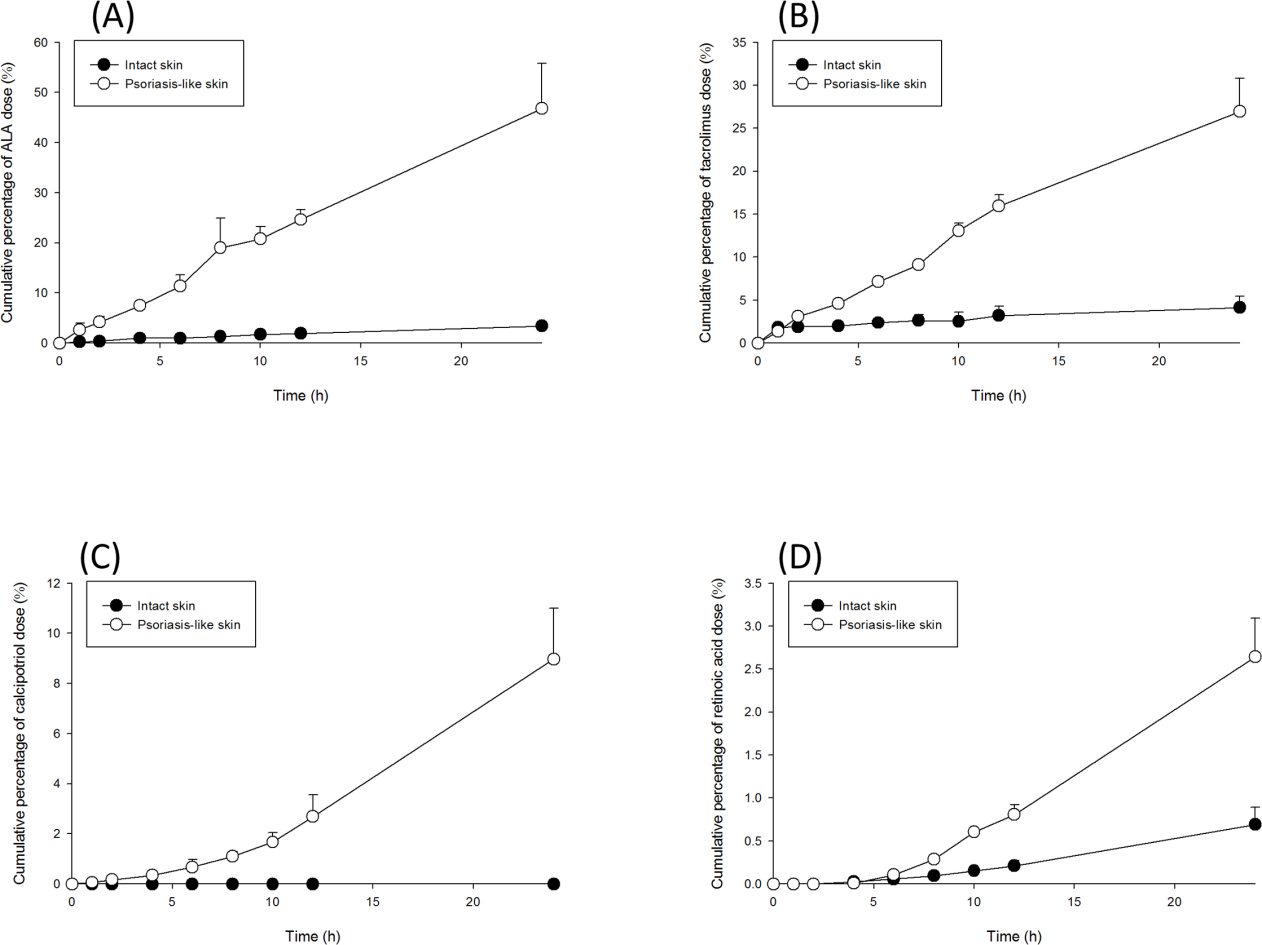
Cumulative percentage-time profiles of drug permeation across intact and psoriasis-like skins: (A) ALA; (B) tacrolimus; (C) calcipotriol; and (D) retinoic acid. The data of are presented as the mean of four experiments±S.D.

Table 2 summarizes the molecular weight (MW) and partition coefficient (log *P*) of four permeants used for testing skin penetration into/across the control and imiquimod-treated skin. The cutaneous delivery of the drugs was assessed using the Franz diffusion assembly method. Both retention in the skin and the permeated amount across the skin were evaluated. The accumulation in the skin indicates cutaneous uptake by topical delivery, whereas the permeated amount predicts transdermal delivery to the systemic circulation. Fig 4 depicts the cumulative percentage-time profiles of the drug dose in receptor compartment. All curves fitted the zero-order fashion. Table 3 shows the penetration data of ALA via control and imiquimod-treated skin. ALA is an extremely hydrophilic permeant with a log *P* of -1.5. The skin deposition of ALA in control skin was 0.18 μg/mg. The amount of the drug accumulated in the skin reservoir for the imiquimod-treated skin was 5.6-fold higher than the control skin. The flux across the psoriasis-like skin was 14.4-fold higher than the control skin. The follicular route, a primary pathway for drug delivery, is used to determine the effect of topical therapies on the appendageal pathway. The amount of ALA deposited in the follicles was evaluated by the combination of stripping and cyanoacrylate casting. The recovery of ALA from the casts is demonstrated in Table 3. After being applied for 24 h, 1.35 μg/cm^2^ of ALA was recovered from the follicles of the control skin. The ALA amount in the hair follicles was found to increase approximately four-fold after being treated with imiquimod.

**Table 2:** Physicochemical properties of the drug molecules tested in this study.

Drug	Partition coefficient (log *P*)	Molecular weight (Da)
5-Aminolevulinic acid	-1.5	167.6
Tacrolimus	3.3	822.0
Calcipotriol	3.8	412.6
Retinoic acid	6.3	300.4

The physicochemical properties of all drugs were obtained from DrugBank website (www.drugbank.ca/ *)*.

**Table 3:** The comparison of skin permeation profiles of 5-aminolevulinic acid, tacrolimus, calcipotriol, and retinoic acid via control and psoriasis-like skins.

Drug	Skin type	Control^a^	Psoriasis-like	Ratio_psoriasis/control_ ^b^
ALA	Skin deposition (μg/mg)	0.18±0.04	1.01±0.11	5.61
	Flux (μg/cm^2^/h)	55.05±8.23	794.28±159.63	14.43
	Follicular uptake (μg/cm^2^)	1.35±0.16	5.17±0.55	3.83
Tacrolimus	Skin deposition (μg/mg)	0.16±0.03	0.13±0.02	0.81
	Flux (μg/cm^2^/h)	2.00±1.04	20.89±3.23	10.45
	Follicular uptake (μg/cm^2^)	1.29±0.16	2.12±0.77	1.64
Calcipotriol	Skin deposition (μg/mg)	0.03±0.01	0.24±0.02	8.80
	Flux (μg/cm^2^/h)	―^c^	1.90±0.34	―
	Follicular uptake (μg/cm^2^)	―	0.91±0.06	―
Retinoic acid	Skin deposition (μg/mg)	0.21±0.05	0.34±0.03	1.66
	Flux (μg/cm^2^/h)	0.19±0.06	0.73±0.12	3.75
	Follicular uptake (μg/cm^2^)	0.42±0.13	1.75±0.52	4.14

^a^ The control group corresponds to the mice treated with the vehicle.

^b^ Ratio_psoriasis/control_, ratio of the permeation parameter of psoriatic skin/ permeation parameter of control skin.

^c^ ―, not determined.

Each value represents the mean and S.D. (*n* = 4).

Tacrolimus is an anti-psoriatic drug with a moderate lipophilicity (log *P* = 3.3). It has a large molecular size with a MW of 822 Da. Tacrolimus levels in the skin between the groups were not significantly different (*p* > 0.05) (Table 3). Nevertheless, imiquimod greatly promoted tacrolimus flux by 10-fold. A greater number of tacrolimus molecules were recovered from the hair follicles of the imiquimod-treated group than the control group; however, this was not a statistically significant difference (*p* > 0.05). Calcipotriol possesses a log *P* (3.8) similar to tarcolimus but has a lower MW (413 Da). In the imiquimod-treated skin the accumulation of calcipotriol was 0.24 μg/mg, which was significantly greater (*p* < 0.05) than that of the control skin (0.03 μg/mg, Table 3). Calcipotriol in the control skin was below the detection limit in the receptor after 24 h. Imiquimod-treated group had a greater calcipotriol flux (*p* < 0.05) across skin compared to the control group. There was no relevant follicular uptake in the control group. Calcipotriol was found to accumulate preferentially in the follicles of imiquimod-treated skin (0.91 μg/cm^2^).

Retinoic acid belongs to the extremely lipophilic drug with a log *P* of 6.3. As shown in Table 3, skin deposition of retinoic acid is significantly higher (*p* < 0.05) for the imiquimod-treated group (0.34 μg/mg) compared to the control group (0.21 μg/mg). The flux across imiquimod-treated skin was 0.73 μg/cm^2^/h, which was approximately four times greater than that across the control group. The imiquimod-treated group had a retinoic acid uptake in the follicles of 1.75 μg/cm^2^, a four-fold increase over the control group.

### In vivo skin distribution of ALA and rhodamine B

**Fig 5:**
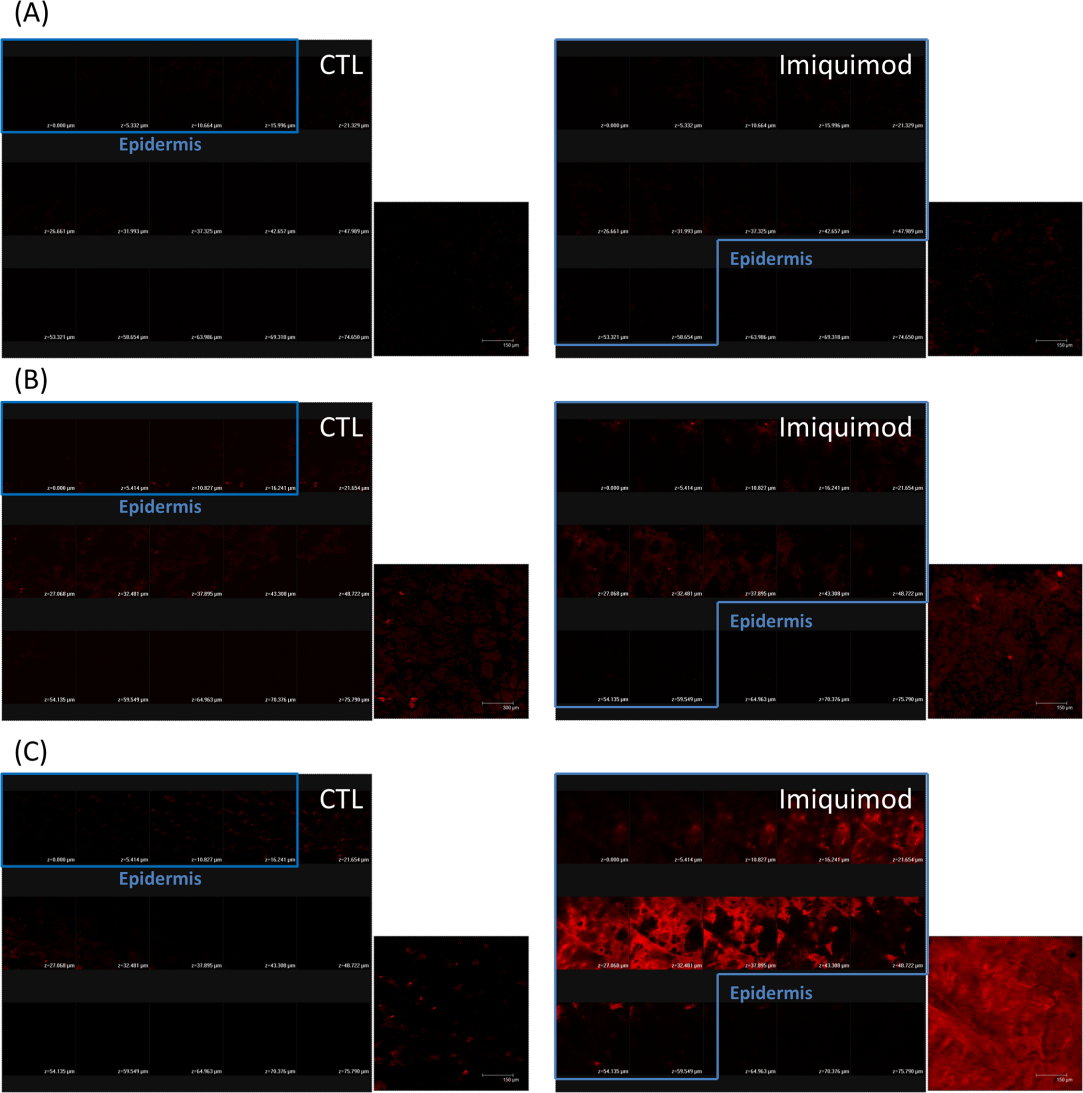
Confocal micrographs of mouse skin treated with and without imiquimod cream: (A) the skin without topical application of permeants; (B) the skin topically applied with ALA; and (C) the skin topically applied with rhodamine B. The control group corresponds to the mice treated with the vehicle. Both the fragments from skin surface to the depth of 75 μm and a summary of 15 fragments at various skin depths are displayed in this figure. Scale bar = 150 μm.

ALA or rhodamine B, a fluorescent dye with a log *P* of 1.95, distributed in the mouse skin was detected following in vivo administration to the dorsal area for both groups. Fig 5 shows the imaging of CLSM where the skin was scanned at ~5-μm increments from the skin surface (left to right, top to bottom). The summary of total 15 fragments is also displayed in Fig 5. Based on H&E histology, the first 4 and 12 fragments could be regarded as the sections of epidermis for control and imiquimod-treated skin, respectively (Fig 2A). It remains to be determined whether the autofluorescence of the skin interfered with the distribution of permeants. As illustrated in Fig 5A, no fluorescence signal is found in the control skin and the imiquimod-treated skin without permeant administration. Fig 5B shows the CLSM images of skin topically applied with ALA. High ALA intracellular concentration can result in biosynthesis of protoporphyrin (Pp) IX to exert red fluorescence. A weak fluorescence was observed in the control skin, with a slightly brighter signal in the upper dermis than epidermis. After ALA application in psoriasiform skin, a slightly higher fluorescence than normal skin was visualized in the summary of fragments. Because of the thickening of the epidermis induced by imiquimod, the fluorescence intensity in the imiquimod-treated epidermis was stronger than that of the control epidermis.

Differing from ALA distribution, rhodamine B distribution is constrained to the epidermal region of normal skin (Fig 5C). Rhodamine B is found to be accumulated within the thickened epidermis of psoriatic skin, as shown in the right panel of Fig 5C. This implied the partitioning of the dye into the damaged SC and epidermis evoked by imiquimod. The fluorescence signal in the imiquimod-treated epidermis was much greater than that in the normal epidermis.

## Discussion

The mouse model was utilized in this study, as it has been useful in simulating psoriasis states [10]. In this study, we confirmed that topical imiquimod on mouse skin can produce human psoriasis-like lesions on phenotypic, histological characteristics and inflammation associated with the IL-23/Il-17 axis. This model revealed an increase in TEWL, a reduction in skin surface pH, desquamation, epidermal hyperproliferation, infiltration of CD3^+^ cells and a decrease in the expression of β-catenin and involucrin on imiquimod-treated skin. The IL-17A, IL-23, and TNF-α also revealed over-expression in this psoriasis-like skin model. It is recognized that the appearance and histology of imiquimod-treated skin was somewhat different from that of van der Fits [10]. No erythema increase was detected in our model, indicating a negligible skin redness. There was a clear presence of granular layer. Although we could observe some erythema in our psoriasis-like skin (Fig 1B), the determination of erythema might be interrupted by the white scaling on skin surface. Thus the increased erythema value was offset by the scaling. However, the close-up imaging of psoriasiform skin exhibited the redness in skin surface. This appearance was similar to human psoriasis, which shows red scaly plaques. The significant presence of granular layer may be due to a common phenomenon accompanied by epidermal thickening although the previous report did not show this evidence. According to the van der Fits’s investigation [10], a fluctuation of cytokine expression is observed during the imiquimod cream treatment period. Our data about mRNA expression of cytokines showed the value after a 8-day treatment of imiquimod. Recently we had carried out the mRNA expression-time profiles of cytokines in the murine model on the formation of psoriatic lesion (unpublished data). The results revealed a transient induction of cytokines such as monocyte chemotactic protein-induced protein 1 (MCPIP1) and IL-19 after imiquimod treatment, which was similar to the experimental results of van der Fits et al. Although these cytokines were reduced at the end of the experiment (8 days), the symptom about psoriatic lesion may be progressive day by day. Further study is needed to elucidate this difference. This study focused on whether psoriasis-like skin induced by imiquimod damaged the skin’s barrier function. The absorption of anti-psoristic drugs via the imiquimod-treated skin was examined in this study. We demonstrated that the drug delivery on the skin deposition and flux increased in imiquimod-induced psoriasis-like skin compared to the control skin. The in vivo study also demonstrated that the drug delivery on the psoriasis-like skin was greater than the control skin using ALA and rhodamine as the markers.

TEWL can be a reflection of skin-barrier function, including the constituents of SC and tight junctions. The experimental results showed an increased TEWL by imiquimod, indicating the disruption of the epidermal barrier by psoriasis. Activated T cells are thought to be the primary modulators of psoriatic pathogenesis. Psoriasis is infiltrated with immune cells, mainly CD3^+^ cells and CD11c^+^ dendritic cells [16]. Our IHC profiles revealed the similarity to human psoriasis with respect to the influx of CD3^+^ lymphocytes in mouse epidermis. Various types of cytokine are involved in the modulation of inflammatory responses in inflammatory skin diseases like psoriasis. Imiquimod has been reported to elicit IL-17A and IL-23 levels in mouse skin, which are features of human psoriasis [17]. Our results proved this upregulation. GM-CSF and TNF-α expression was also increased by imiquimod in the present work. IL-17A can activate keratinocytes to release cytokines and antimicrobial peptides, contributing to the inflammatory and immune processes [18]. It acts synergistically with TNF-α, exhibiting some chronic disease features [19]. TNF-α plays a primary role in activating adhesion molecule expression of vascular endothelial cells for trafficking lymphocytes and leukocytes in psoriatic region [20]. This result was in line with the CD3^+^ cell infiltration in psoriatic lesions. The previous report [21] has demonstrated that CD3^+^ cells in psoriasis upregulated the expression of IL-23. IL-23 induces several psoriasis features such as acanthosis and erythema [10]. IL-23 and psoriatic CD3^+^ cells also promote GM-CSF production, stimulating the growth of inflammation-related granulocytes such as neutrophils and eosinophils [22].

IL-17, IL-23 and GM-CSF are involved in the T-helper (Th)17 cell response [23,24]. Th1 immune response is another possible pathway in the triggering of plaque psoriasis. IL-12 is a potent inducer of the Th1 differentiation with naïve lymphocytes becoming IFN-γ-producing cells [25,26]. Our results showed that imiquimod did not increase IL-12 and IFN-γ in mouse skin. This may indicate a predominant association between psoriasis and Th17, but not the Th1 pathway in our case. A recent report [27] confirmed that the inflammatory cascade of psoriasis is attributed to IL-23 rather than IL-12.

IL-17 can raise the expression of antimicrobial peptides and decrease cell adhesion factors, thus impairing the skin barrier function [19]. We further checked the possible alteration of the skin barrier function by imiquimod intervention. The biomarkers examined by IHC or qRT-PCR included β-catenin, filaggrin and involucrin. The experimental profiles exhibited a downregulation of involucrin, but not filaggrin. Moreover, β-catenin was absent in the upper epidermis of imiquimod-treated skin although this protein was still abundant in the lower epidermis. β-catenin plays a role in the regulation of intercellular mobility; it is fundamental to maintain epidermal junctions under externally applied stress [28]. Previous study [29] demonstrate β-catenin expression in human keratinocyte nuclei of psoriatic lesion. It is not the case in our mouse model. Recent studies [30,31] have demonstrated the decreased β-catenin in the lesional skin of psoriatic patients, suggesting a reduction of cepidermal barrier effect. It should be noticed that although the downregulation of β-catenin in the upper epidermis of imiquimod-treated skin could elicit deficient barrier function, the total amount of β-catenin in epidermis might not be reduced compared to intact skin in terms of the increased number of keratinocyte layers. Involucrin is a cornified-envelope structural protein that dominates barrier function [32]. Moreover, it is an indicator of keratinocyte differentiation [33]. Filaggrin is a structural protein, which is postulated to participate in the mechanical strength and integrity of SC and epidermal barrier function [34]. It is present in both SC and stratum granulosum. Imiquimod intervention did not downregulate the expression of filaggrin. The presence of granular layer after imiquimod treatment confirmed the existence of profilaggrin in the psoriasiform skin.

According to the skin penetration profiles, most of the permeants could facilely diffuse into/across psoriasiform skin compared to healthy skin. This indicates that the barrier function defect, and not elongation of delivery pathlength, dominates drug absorption through psoriatic skin. The full epidermis, including SC and epidermal junctions, offers a permeability barrier for drug diffusion. Psoriasis-induced barrier impairment occurs via SC disruption according to TEWL increment. The barrier was also weakened by the loss of β-catenin and involucrin. These deficiencies are momentous factors in the enhancement of drug absorption via psoriatic skin.

The SC is proved to be a critical permeation barrier for ALA [35]. SC damage by imiquimod treatment led to a great enhancement of ALA skin deposition and flux. ALA displayed a significant accumulation in the receptors because the hydrophilic permeant can be forced into lipophilic skin compartments, and then immediately continue to the hydrophilic receptor medium [36]. Smits et al. [37] demonstrated a 4-fold PpIX increment in lesional skin of psoriasis as compared with normal skin, which approximated the ratio_psoriasis/control_ of skin deposition in our study (5.6-fold). Topically-applied tacrolimus has been verified to show poor absorption and inefficient therapy in regard to psoriatic lesions due to the thick plaques [38]; our results confirmed this result since imiquimod intervention did not further increase tacrolimus accumulation in skin reservoir. However, tacrolimus flux was notably increased by imiquimod treatment. This may be due to the saturation of the skin reservoir by tacrolimus. Further skin disruption by psoriasis could not increase tacrolimus deposition because of the limited capacity for drug loading, but did largely penetrate across the skin into the receptors.

Calcipotriol could locate within normal skin without any penetration into receptors after topical application. This result was consistent with clinical observation that calcipotriol has very low systemic absorption [39]. The treatment of skin by imiquimod promoted calcipotriol penetration into receptors, which may result in the increased risk to systemic circulation. Retinoic acid also showed a low flux in normal skin. It is difficult for the lipophilic retinoic acid passing through the hydrophilic viable skin to approach the receptors [14]. Viable epidermis/dermis provides an important resistance for extremely lipophilic drugs since the partitioning into aqueous resistance is not easy for these permeants [6]. Barrier disruption by imiquimod moderately increased retinoic acid deposition and flux, indicating the importance of epidermal junctions in the obstruction for lipophilic drug absorption. The enhancement level by imiquimod for retinoic acid permeation was significantly lower than that for ALA, the extremely hydrophilic drug. This suggests that SC still possesses the main role in controlling cutaneous permeation, though the role of epidermal junctions cannot be neglected.

The steady-state flux of the permeants across skin can be influenced in terms of lipophilicity and molecular size [40]. For the four drugs tested in this study, the more hydrophilic permeants exhibited higher flux enhancement after imiquimod treatment due to SC damage. This indicates a correlation between flux across psoriasis-like skin and log *P*. The percutaneous absorption of more hydrophilic drugs would be more affected by the skin with compromised barrier function. Tight junctions can efficiently prevent the diffusion of permeants with a MW of > 600 Da [41]. Barrier damage in a pathological condition allows more effortless penetration of the permeants, especially for the large molecules [34]. Imiquimod increased tacrolimus (822 Da) flux by a factor of 10 compared to the normal skin. However, this enhancement did not surpass ALA with a smaller size (168 Da). This may suggest a limited impact of molecular volume on the drug permeation via psoriasis-like skin.

The follicular density in skin varies depending on the sites on the body, with the scalp holding the highest density [42]. On the other hand, the largest diameter of the follicular opening is observed in the calf [43]. Psoriasis highly occurs in the regions of scalp and limbs. The follicles occupy 10% of the total scalp area. In addition, the scalp skin appears to be more permeable than the other anatomic sites [44]. The hairy mouse may be especially suitable as the alternative for scalp skin to examine drug delivery into follicular pathways. The follicular route may render a significant access to drugs penetrating psoriatic skin. For calcipotriol and retinoic acid, the two most lipophilic permeants, the uptake by follicles was less than ALA and tacrolimus in normal skin. This was in line with the investigation by Frum et al. [45] that the drugs with higher lipophilicity show a minor entrance into follicles. ALA is preferred in the absorption by hair follicles, the aqueous pathway of skin [46]. The follicular uptake of ALA could be further increased by imiquimod treatment. The follicular epithelium exhibits less resistance than interfollicular epithelium [42], providing a fast and easy way to transport the drug into deeper skin strata and even systemic uptake. This can partly explain the great increment of ALA flux by imiquimod application. Retinoic acid also showed a strong selectivity for hair follicles after imiquimod treatment, indicating the impact of psoriasis on increasing retinoic acid delivery into appendages.

We further explored *in vivo* cutaneous distribution of ALA and rhodamine B. It can be seen in the CLSM image that the PpIX level in psoriasis-like epidermis was higher than that in normal epidermis; the facile ALA penetration into defective skin barrier was responsible for this increment. Another possibility is the reduced ferrochelatase activity and increased porphobilinogen deaminase by the hyperproliferative tissue [47], which encourages PpIX production. A limited amount of rhodamine B penetrated the epidermal layer of intact skin. A same phenomenon was demonstrated by Andrews et al. [3], who suggested a significant penetration barrier of both SC and viable epidermis for this dye. There was a great augmentation in skin permeation to rhodamine B after imiquimod treatment, indicating the role of SC and epidermal junctions in promoting drug diffusion via psoriasis-like skin.

Besides the therapeutic efficacy of psoriasis treatment modalities, an important concern is the drug tolerance and the safety of the patients. PDT with ALA causes skin edema and inflammation in some cases due to direct damage to fibroblasts and mast cells [48]. The common adverse effects of topical tacrolimus on skin are itching, burning and erythema. About 20% of psoriatic patients undergo cutaneous irritant reactions after topical application of calcipotriol [49]. The adverse effects of retinoic acid such as erythema, burning, desquamation and stinging are concentration-dependent [50]. The occurrence of these toxic risks is partly due to the over-absorption of anti-psoriatic drugs in the skin with a deficient barrier characteristic. The action target for anti-psoriatic drugs is mainly in the epidermis. The permeation profiles revealed that imiquimod increased flux to a higher level rather than the increased ratio of skin deposition. The possible toxicity in circulation should be a concern of this over-absorption. Although currently there is no evidence signifying the risk of anti-psoriatic drug over-absorption, long-term monitoring and the modulation of the applied dose may be suggested. The topical administration on large areas of the diseased skin should also be avoided. According to the data of the National Psoriasis Foundation of USA, the annual direct and indirect costs of psoriasis in USA are over $11 billion in US dollars [7]. The dose reduction and risk control of the anti-psoriatic drugs can lessen the expense for treating psoriasis.

Another concern is that most of the investigations have employed normal skin for assessing percutaneous absorption of the drugs. This can underestimate the real absorption in the diseased skin. Some attempts such as penetration enhancers, iontophoresis, microneedles and nanocarriers are undertaken to enhance ALA flux for successful delivery into target sites [51]. Normal skin is frequently used as the permeation barrier to test the usefulness of the enhancing techniques. For example, ALA flux is found to increase 2.4-fold upon the insertion of microneedles in normal mouse skin [46]. Zhang et al. [52] indicated a 2.6-fold increase in ALA flux after formulating this drug into nanoemulsions. Our results showed a 14.4-fold increase of ALA fluxs in the case of psoriasis-like skin compared to normal skin. From the practical viewpoint it is important to employ diseased skin as the barrier for testing drug delivery. This can simulate the actual condition in the clinics.

Although a successful result was achieved in this report, it should be cautious to reflect the clinical condition by these data using the mouse as the animal model. The mouse skin is thinner and more-permeable as compared to human skin. Though the porcine skin is a well-recognized alternative for human skin [53], there is still no porcine skin model developed for simulating psoriatic lesion. Whether or not the results from mouse can be correlated with human needs further examination. Another limitation of this study is that we believe there were some discrepancies between skin physiology and histopathology of the experimental psoriasis-like skin and the psoriasis skin in humans. The most different feature between experimental and human psoriasis is the granular layer. The hypogranulosis formed in human psoriasis was absent in the mouse model tested in the present work. Another difference can be the reduced filaggrin expression in human psoriasis but not in experimental psoriasis [54]. Filaggrin is mainly present in SC and granular layer. Because of the maintenance of granular layer in psoriasis-like mouse skin, filaggrin expression was not altered in our model. The induction of psoriatic lesion in animals is based on local stimulation, whereas the psoriasis in humans results from the modulation of systemic immune response. Swindell et al. [55] had compared the genome-wide expression profiles of five mouse models mimicking psoriasis, including imiquimod-treated and transgenic mice. They suggest that each mouse phenotype exhibit some variations in epidermis- or keratinization-associated expression patterns compared to human psoriasis. The animal model utilized in the present work was unique and not the real psoriasis, in spite of its similarity to psoriatic lesion in many aspects as the experimental results shown.

## Conclusions

This study demonstrated that imiquimod-induced psoriasis-like skin in mice is an effective model for investigating drug activity. Our findings demonstrated the increased skin deposition and flux of the drugs by psoriasis-like skin as compared to healthy skin. The drug lipophilicity was an essential factor controlling permeation enhancement by imiquimod. The most pronounced enhancement was observed with hydrophilic drugs. The imiquimod treatment did not largely alter skin accumulation and the flux of lipophilic permeants. We found that SC disturbance by imiquimod dramatically promoted cutaneous drug absorption. Most investigations have used intact skin for evaluating drug absorption. The results from such research may not be feasible for predicting cutaneous delivery of the drugs on diseased or damaged skin. The skin model used in this work is beneficial for resolving this issue, especially the anti-psoriatic activities.

## Supporting Information

S1 Text The physiological parameters, gross appearance, and histology of the mouse skin without any treatment. (DOCX)
