# Supplementary material for: Using Imiquimod-Induced Psoriasis-Like Skin as a Model to Measure the Skin Penetration of Anti-Psoriatic Drugs
Source: PLoS One. 2015 Sep 10;10(9):e0137890. doi: 10.1371/journal.pone.0137890 (PMC4565663; doi:10.1371/journal.pone.0137890)

Table. Transepidermal water loss (TEWL) and erythema values of the mouse skin without any treatment

| TEWL (g/m^2^/h) | Erythema |
| --- | --- |
| 6.72±3.52 | 3.23±1.02 |

Figure. Gross appearance and H&E (x200) of the mouse skin without any treatment


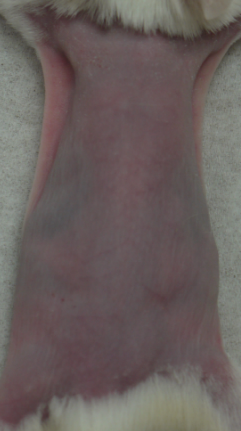


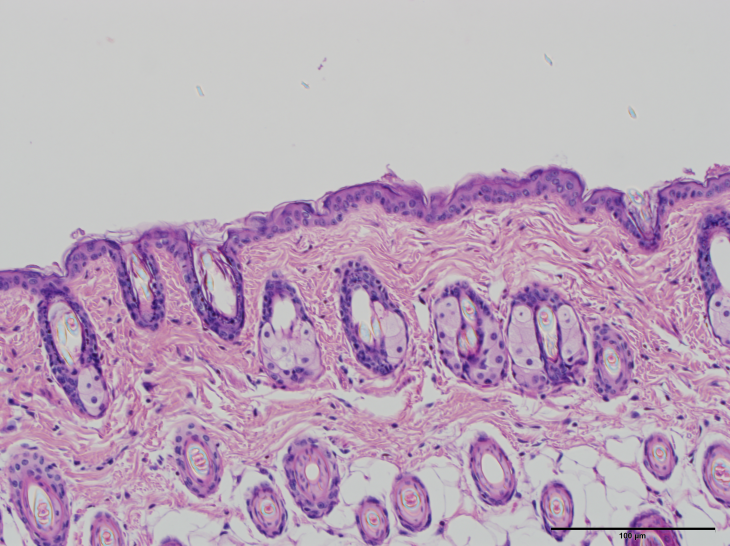

Supplement: S1 Text — (DOCX) [file pone.0137890.s001.docx]
